# Promoting universal financial protection: a case study of new management of community health insurance in Tanzania

**DOI:** 10.1186/1478-4505-11-21

**Published:** 2013-06-13

**Authors:** Josephine Borghi, Stephen Maluka, August Kuwawenaruwa, Suzan Makawia, Juma Tantau, Gemini Mtei, Mariam Ally, Jane Macha

**Affiliations:** 1 London School of Hygiene and Tropical Medicine, 15-17 Tavistock Place, London WC1H 9SH, UK; 2 Ifakara Health Institute, Kiko Avenue, Plot 463, Kiko Avenue Mikocheni, P.O. Box 78373, Dar es Salaam, Tanzania; 3 Institute of Development Studies, University of Dar es Salaam, P.O. Box 35131, Dar es Salaam, Tanzania; 4 District Council, Singida Rural, Ministry of Health and Social Welfare, Singida, Tanzania; 5 Department of Policy and Planning, Ministry of Health and Social Welfare, Samora. Avenue/Shaban Robert Street Junction Plot no: 36/37, P.O. Box 9083, Dar es Salaam, Tanzania; 6 Department of Global Health and Development, Faculty of Public Health and Policy, London School of Hygiene and Tropical Medicine, 15-17 Tavistock Place, London WC1H 9SH, UK

**Keywords:** Financing, Health insurance, Informal sector, Merger, Reform, Tanzania

## Abstract

**Background:**

The National Health Insurance Fund (NHIF), a compulsory formal sector scheme took over the management of the Community Health Fund (CHF), a voluntary informal sector scheme, in 2009. This study assesses the origins of the reform, its effect on management and reporting structures, financial flow adequacy, reform communication and acceptability to key stakeholders, and initial progress towards universal coverage.

**Methods:**

The study relied on national data sources and an in-depth collective case study of a rural and an urban district to assess awareness and acceptability of the reform, and fund availability and use relative to need in a sample of facilities.

**Results:**

The reform was driven by a national desire to expand coverage and increase access to services. Despite initial delays, the CHF has been embedded within the NHIF organisational structure, bringing more intensive and qualified supervision closer to the district. National CHF membership has more than doubled. However, awareness of the reform was limited below the district level due to the reform’s top-down nature. The reform was generally acceptable to key stakeholders, who expected that benefits between schemes would be harmonised.

The reform was unable to institute changes to the CHF design or district management structures because it has so far been unable to change CHF legislation which also limits facility capacity to use CHF revenue. Further, revenue generated is currently insufficient to offset treatment and administration costs, and the reform did not improve the revenue to cost ratio. Administrative costs are also likely to have increased as a result of the reform.

**Conclusion:**

Informal sector schemes can benefit from merger with formal sector schemes through improved data systems, supervision, and management support. However, effects will be maximised if legal frameworks can be harmonised early on and a reduction in administrative costs is not guaranteed.

## Background

During the last ten years, Tanzania has made efforts to expand health insurance coverage. However, health insurance remains fragmented [1], and coverage is low. Currently the two largest health insurance schemes are the National Health Insurance Fund (NHIF), a mandatory scheme offering comprehensive benefits to the formal sector, and the Community Health Fund (CHF), a voluntary scheme for the informal sector in rural areas, offering limited benefits in public lower level facilities. While NHIF coverage has been gradually increasing since its introduction, CHF coverage has remained low due to weak management, poor understanding of the concept of risk pooling [2], and a limited benefit package [3].

In early 2009, the NHIF took over the management of the CHF from the Ministry of Health and Social Welfare (MoHSW) initially for a 3 year period, a first step towards the merger of these schemes. The merger of insurance schemes, particularly schemes targeting the informal and formal sectors, is a precursor to the development of social health insurance [4], which has been considered by a number of countries in Africa [5,6]. The linkage of insurance schemes has also been recommended as a means of strengthening informal sector scheme management and administrative capacity [7,8], reducing administrative overheads [9], increasing pooling [9] and coverage [10], and achieving universal coverage [11]. However, there is limited published data regarding the process of merging schemes. Evidence from Asia and Latin America indicate this can present challenges including the need for benefit package convergence, dealing with adverse selection and ensuring financial sustainability [12,13].

A number of factors can enhance the effectiveness of health financing reforms more generally, including awareness [14-16] and acceptability of the reform among key stakeholders [17], and adequacy of financial resources and management systems.

The Tanzanian context provides a unique opportunity for looking more closely at the merging of a formal and informal sector scheme, to assess if and how this might enhance progress towards universal coverage. The objectives of this paper are to ascertain to what extent the initial motivation for the reform was driven by a desire for progress towards universal coverage; assess the impact of the reform on management and reporting structures; assess the responsiveness of and adequacy of financial flows; describe how the reform has been communicated to key stakeholders and assess its acceptability; examine if the reform has made any initial progress towards universal coverage in terms of pooling and purchasing.

Although the reform is still at an early stage, this paper assesses whether the foundations are in place for progress in terms of universal coverage.

### Cost sharing in Tanzania

The NHIF and the CHF were officially introduced in 2001. User fees in public lower level facilities were introduced alongside the CHF, along with a system of exemptions (free care for priority population groups (e.g., children under five, pregnant women) and waivers (free care to those who are unable to pay). The NHIF is mandatory for public servants, with other formal sector employees being able to opt into the scheme. The NHIF is funded by a 6% payroll contribution, split evenly between the employer and employee, and covers the contributor, their spouse and up to four additional legal dependants. Benefits include inpatient and outpatient care at all public facilities and accredited private and faith-based facilities and pharmacies. Providers are reimbursed on a fee-for-service basis. The NHIF is administered by an independent body answerable to the MoHSW.

About 90% of the population in Tanzania are in the informal sector [18]. The CHF is a voluntary pre-payment scheme targeting the informal sector. The scheme was rolled out by the MoHSW, with financial support from the World Bank. Households can enroll for between USD 4 to 8 per year. Benefits include free outpatient care at a selected primary level public facility. Providers are not reimbursed for use of services by members, but can use CHF revenue to purchase drugs, medical supplies, equipment, furniture, and facility maintenance and certain allowances [19]. A few districts also cover some costs of inpatient care at referral facilities [3]. Subject to the district submitting requests, the central government will match the contributions made by CHF members through a matching grant.

## Methods

Information on the origins of the reform and management structures was compiled through a review of relevant policy documents combined with in-depth interviews with national stakeholders from the MoHSW (n = 2); former national CHF coordinators (n = 2); members of parliament (n = 2); NHIF staff (n = 2); and donors (n = 4). Data obtained from different sources were triangulated for validation, in the rare cases where inconsistencies were identified, further follow-up questions were administered to the relevant respondents by telephone. Information on national financial flows and insurance coverage were obtained from reports and data at the MoHSW and the NHIF.

The study adopted a multiple case study design whereby we undertook in-depth data collection from a small sample of purposively selected case study sites. Two sites were chosen to be able to compare and contrast potential similarities and differences in experiences of the reform arising in different settings. The focus was on awareness and acceptability of the reform to different stakeholders, and fund availability and use relative to need at facility level.

### Data collection and analysis methods

#### District context

The case study districts were selected in consultation with the NHIF, such that the districts had a number of years of CHF experience, and a reasonable number of CHF members. It was decided to sample districts from the same region, to control for geographic variation, and for ease of access. A rural and an urban district were purposively selected to compare reform experience in districts with different CHF histories, as well as different financial management systems, and benefits available to members. Populations in urban councils in many regions of the country are largely comparable to those of rural districts. The distinguishing feature of urban settings that was pertinent to this study was the more recent introduction of the CHF concept, which was originally designed for rural districts. The rural district had over 10 years’ experience implementing the CHF and good district records, whereas the urban district had introduced CHF in 2008 (Table 1). User fees were in place in all public facilities in the two districts. Procedures for accessing cost sharing funds which includes user fees, CHF revenue, and NHIF reimbursements varied across the two districts. In the rural district, cost sharing funds were pooled at the district level in a ‘CHF account’. To use cost sharing funds, facilities would send a request to the district. The amount of drugs and supplies purchased for a facility is not tied to the amount of cost sharing revenue generated by the facility, leading to cross subsidization between facilities.

**Table 1:** Selected characteristics of sampled case study districts

**Characteristics**	**Rural district**	**Urban district**
Population size	486,900	175,717
Proportion of the population living below the poverty line (%)	56	46
Number of health facilities	57	16
Number of government facilities	47	10
Population per health facility	8,542	10,982
Year of introducing CHF	1999	2008
Number of CHF member households and estimated population coverage (2011)	11,802 (12%)	792 (2%)
CHF premium	3.13 USD per household per year	3.13 USD per household per year
CHF benefit package	Outpatient care in selected public primary facility (dispensary or health centre) plus referral care up to 9.38 USD in regional hospital, district designated hospital (faith-based)	Outpatient care in selected public primary facility (dispensary or health centre)
User fee level	0.63 USD until 2009	0.63 USD for dispensaries and health centres
	1.89 USD since 2009	
Financial flows	CHF, NHIF and user fee revenue pooled in district CHF-account	CHF and user fee revenue deposited in facility bank account. NHIF revenue pooled in the District Medical Officer account

Source: Comprehensive Council Health Plans of respective districts, 2010/2011 [20].

In the urban district, facilities have their own bank accounts since 2007/2008 and the CHF and user fee funds are deposited directly into this account and can be spent without district approval. There is no district level ‘CHF account’ allowing for district level risk pooling and no cross-subsidisation between facilities.

The rural district had contracted the regional hospital, the district hospital (a faith-based hospital) and, at the time of the study, was in the process of entering into an agreement with a second faith-based facility in order to provide inpatient care to its members up to the value of USD 9.38. In contrast, the urban district did not offer referral services to its CHF members.

### Qualitative methods

District level data were collected in May and August 2011, and February 2012. Stakeholders were selected purposively in order to ascertain the impact of the reform on management structures as well as levels of awareness of the reform. Interviews were, therefore, conducted with stakeholders with experience managing or running the CHF and with insurance beneficiaries. A total of 33 interviews were carried out. Interviews were conducted with the district CHF manager in each district, a sample of health workers who mobilise people to join the CHF and enroll CHF members from four health facilities (described below) and with health facility governing committee members responsible for overseeing resource use at facility level including CHF revenue from these sampled facilities (16 in the rural district, 17 in the urban district), and six national level interviews were conducted including the current CHF director at the NHIF and the former CHF national coordinator. Additionally, seven focus group discussions (FGDs) were conducted, three in the urban district and four in the rural district (three with CHF members and four with uninsured individuals, with each focus group including between eight to 12 participants). Two FGDs were conducted with members of the NHIF at two government secondary schools in Ilala district of Dar es Salaam. Five members participated in the discussion at the first school and two members participated in the second school. All interviews and FGDs were conducted in Kiswahili by two social scientists. The data were transcribed verbatim and translated by support staff, and the translated data were subsequently checked by the social scientists. Data were classified and manually coded according to key themes guided by the research questions using thematic content analysis. Analysis was first conducted for each case study site separately. Thereafter the findings from each site were compared and contrasted.

### Quantitative data

Data were collected from a purposive sample of four health facilities: a public dispensary and a health centre in each of the districts. The district CHF coordinator guided the final choice of facilities. The criteria for selection included geographical accessibility, at least 100 CHF members, and the availability of a health worker in-charge of the facility who would be supportive to the research team.

Information on facility cost sharing revenue and expenditure were compiled for the years 2008 to 2011 from each of the facilities. CHF revenue was compared to the costs of treating CHF members and managing the CHF at facility level (encouraging people to join; enrolling members; issuing cards; managing funds). To estimate treatment costs, unit costs were multiplied by the number of outpatient visits and admissions (in the rural district) for CHF members. Average reimbursement rates for outpatient visits and inpatient admissions by the NHIF were used as a proxy for unit costs. The number of visits/admissions by CHF members was extracted from facility registers. In some facilities, reported service use by CHF members was surprisingly low (less than one visit per household per year), raising concerns about data reliability. Hence, we also used data collected within the SHIELD study^a^ on the average annual number of outpatient visits among CHF members (an average of 7.4 visits per household per year, assuming 5 individuals per household) [21]. CHF management costs were estimated by interviewing those involved in CHF administration at the facility level, and reflected current management practices as of 2010/2011 [22]. We were unable to measure eventual changes in administration costs resulting from the reform. Quantitative data were compiled and analysed using Microsoft excel. All costs are presented in USD using the exchange rate 1,600 TSH to 1 USD.

Ethical approval for the study was obtained from the Institutional Review Board of the Ifakara Health Institute in Tanzania, and from the Ethics Review Committee at the World Health Organisation, in Geneva.

## Results

### Origins and rationale for the reform

On 4th June 2009, a Memorandum of Understanding (MOU) was signed by the NHIF, the MoHSW, and the Prime Minister’s Office for Regional Administration and Local Government (PMO-RALG) giving management responsibility for the CHF to the NHIF for a 3 year period. The decision to place the management responsibility for the CHF with the NHIF was guided by the MoHSW’s objective of increasing national health insurance coverage set out in the Health Sector Strategic Plan [23], and a range of other factors (Table 2). A donor funded workshop in 2007 highlighted weaknesses in CHF management and constraints to coverage expansion [24,25] resulting in the commissioning of a ten year health sector evaluation, which recommended synchronizing the NHIF and CHF [26]; which was formalized in a Cabinet directive to the MoHSW. In 2008, the merger of the two schemes was included as a scenario in a regulatory framework feasibility assessment [27]. The fact that both the NHIF and the CHF report to the MoHSW also facilitated the linkage of the two schemes [28]. There also appeared to be close alignment between donor and government interests in seeking to expand coverage through harmonization of schemes.

**Table 2:** Chronology of events preceding and following the takeover of the management of the CHF by the NHIF

**Date**	**Event**
2007	Cabinet directive No 37/2007 to synchronise the NHIF and the CHF to support the implementation of the Primary Health Services Development Programme and provide technical and managerial support and extend CHF coverage.
31st January- 2nd February, 2007	CHF best practice workshop in Dar es Salaam funded by SDC and GTZ in collaboration with the MoHSW.
October, 2007	Ten year evaluation of the health sector recommending synchronization of NHIF and CHF operations conducted by an external consultant and commissioned by development partners and the Government of Tanzania.
March, 2008	Resolution by MoHSW management team that the NHIF should oversee CHF.
August, 2008	Regulatory framework feasibility study commissioned by the MoHSW which included a scenario on merging the NHIF and CHF funded by GIZ and SDC.
4th June, 2009	Signing of MOU between the MoHSW, PMO-RALG and NHIF management. Secondment of MoHSW national CHF coordinator to the NHIF.
September 2009	Country evaluation of the net worth of the CHF by the NHIF.
October 2009	The CHF action plan 2009–2012 developed.
February 2010	Appointment of staff to oversee CHF at the zonal/regional NHIF offices.
March 2010	The NHIF began payment of matching funds to the districts.
September 2011	CHF Directorate created within the NHIF.
Late 2011	CHF action plan revised for remaining year.
December 2011	Meeting of CHF coordinators from across the country to inform about the reform and set targets to meet 30% coverage nationally.
February-April 2012	National client days to gather opinions on CHF implementation.
	Printing and distribution of CHF leaflets and posters.
	Districts are instructed to budget for use of CHF cars that can be used to promote CHF, showing promotional films to communities.
June 2012	NHIF and CHF management teams expected to report on 3 year experience and present plan for coming years to the MoHSW and PMOLARG.

The MOU objectives were to harmonise NHIF and CHF management operations by incorporating CHF management structures within NHIF, to improve efficiency and supervision, to increase awareness of the CHF, and to increase coverage in line with universal coverage objectives [29].

*“The government felt that the NHIF has strong experience in managing a health insurance scheme, it has experts, and it has many zonal offices.*” (National level respondent)

The MoHSW were to cover the recurrent costs of managing the scheme along with the matching grant funds. The reform was also intended to improve access to services by providing support to the Primary Health Services Development Programme (known locally as the ‘MMAM’) which was designed to bring health services closer to the population. However, there is no evidence to suggest that the reform was explicitly intended to increase financial protection.

Shortly after the MOU, the NHIF conducted a status assessment to determine national CHF coverage and developed a three year action plan [30]. An estimated USD 13.1 million were requested from the MoHSW to cover the costs of running the CHF for the three year period. Delays in approval of the action plan and failure to secure all of the requested funds delayed reform implementation. In 2010, districts began claiming matching funds from the NHIF, a review of the claiming process was conducted, and changes to CHF management systems were introduced. In 2011, a revised action plan was prepared with a more limited set of actions to be completed by 2012. In 2011 a CHF Directorate was created within the NHIF. Later that year a national consultation meeting with CHF coordinators from across the country was organised by the NHIF to inform them of the reform and its objectives as well as to emphasize the need for expanding CHF coverage; a nationwide information campaign was launched to expand CHF enrolment.

### Management structures pre- and post-reform

**Figure 1:**
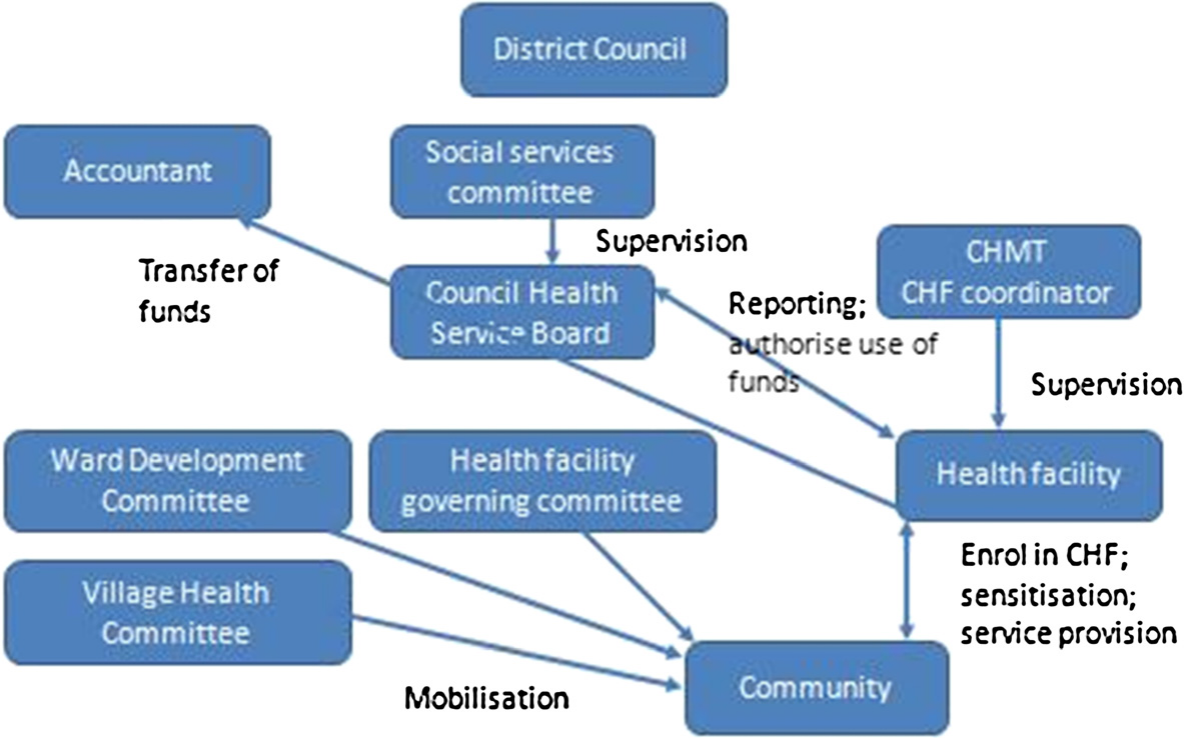
Overview of management structures from the district level down.

The CHF, at district level, is under the management of the Council Health Service Board (CHSB) which comprises medical professionals and community representatives (Figure 1). There is one CHSB per district, covering the entire district population. The CHSB must be established by the district when introducing the CHF to oversee cost sharing fund management and use at the district level. A district CHF coordinator is responsible for tracking membership levels and reporting on CHF and user fee funds collected by the district. This person typically has another full time role. Since taking over the CHF, the NHIF are encouraging districts to employ this person full time. In 2011 the coordinator in the rural district became a full time coordinator, but not in the urban district. Further, no additional resources have been offered to facilitate this process. The design of the CHF (in terms of premiums and benefit package) is specified in a by-law that is drafted by district managers and approved by community groups and ultimately the Prime Minister’s Office. At the ward level, the Ward Health Committee and the Health Facility Governing Committee along with health workers in the facility have responsibility for mobilising people to join the CHF (Figure 1). They are also responsible for informing the community about the levels of funds raised and how funds are used within the facility [31].

The reform has not led to any changes in the district level structures as these are stipulated within the CHF Act, and cannot be changed without a change in legislation, which was not expected within the current MOU. Further, the NHIF are not yet embedded within district management structures, as the CHSB does not include NHIF staff. The reform has, however, led to significant changes in national and zonal/regional level management systems.

**Figure 2:**
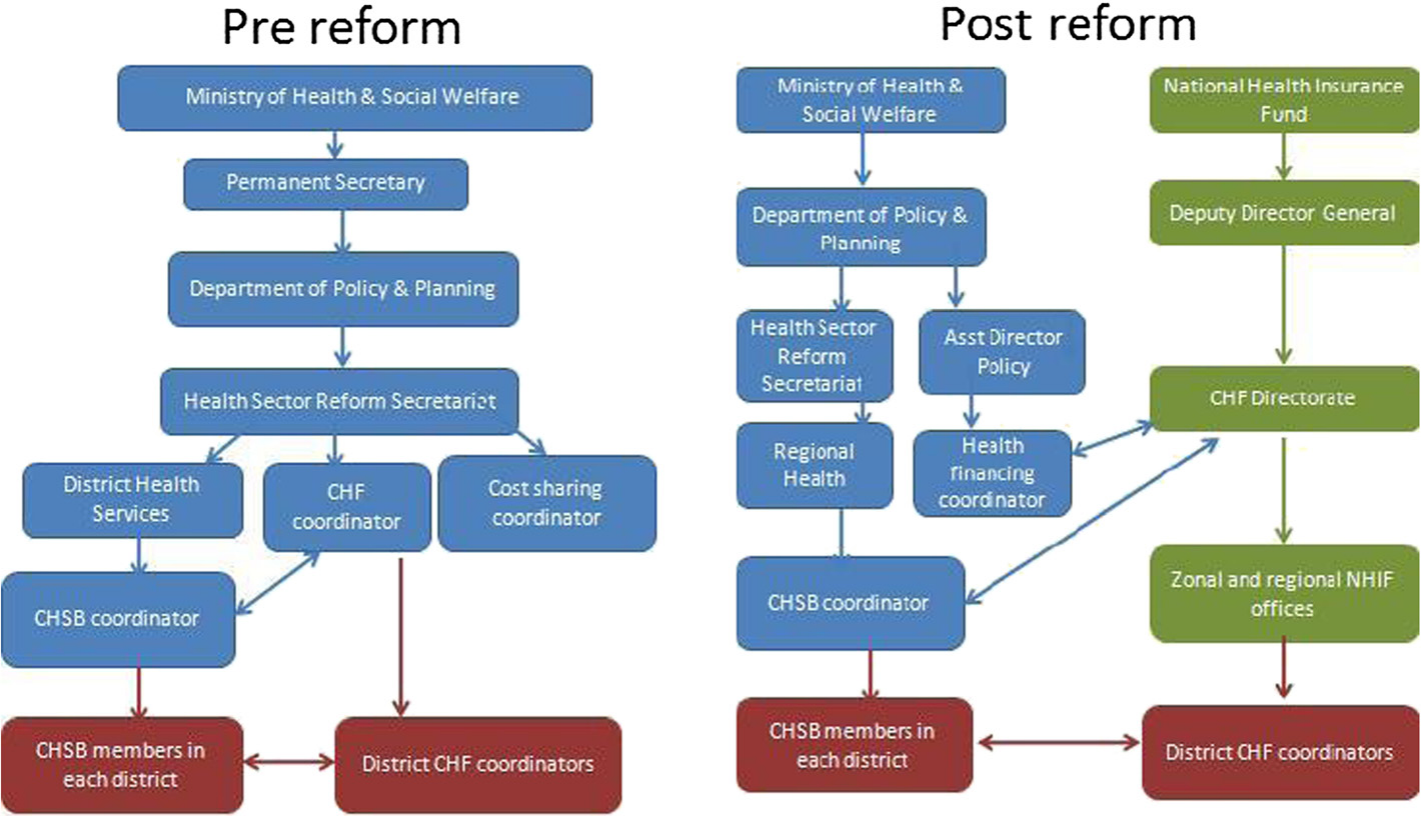
Overview of management structures from the central to district level before and after the reform.

Prior to the reform, the national CHF coordination unit sat within the MoHSW, and was headed by a coordinator supported by two assistants, who undertook training of district managers on CHF, and oversaw the enactment of the bylaws for the CHF (Figure 2). District level supervision visits typically took place once per year, and CHF performance was also assessed during regional management meetings which took place annually. Stronger CHF coordinators would support weaker ones through *ad hoc* meetings with the latter. Matching fund requests were channelled to the national CHF coordinator from the districts, along with reports of membership/enrolment, which were often incomplete. There was no national data on CHF coverage. Data were only available for matching grant disbursements and only a limited number of districts claimed for the matching fund due to insufficient membership (a minimum of USD 3,125 was required before a claim could be made) and/or a lack of awareness of the claiming procedure.

Since the reform, a CHF Directorate within the NHIF headed by a CHF Director, reporting to the Deputy Director of the NHIF and supported by a team of seven people, oversees CHF operations centrally. The CHF Director also reports to the Health Financing Coordinator at the MoHSW (Figure 2). NHIF staff in 13 NHIF zonal and regional offices have been appointed to support district CHF coordinators across the country. There are plans to introduce NHIF offices in all regions of the country. Matching fund requests and payments are managed by the NHIF zonal/regional offices, although funds themselves are still provided by the government.

The national CHF coordinator (pre-reform) and the CHF Director (post-reform) work closely with the national CHSB coordinator who supports to the establishment of the CHSB within the district, and the development of the district by-laws.

CHF supervision is now integrated into the routine visits by NHIF zonal or regional staff which typically take place once per month or per two months. During these visits, officers check on CHF and matching grant reporting at district level and sometimes visit facilities to check drug availability, hence providing a quality assurance role to CHF members.

*“At the moment we regularly go with NHIF zonal coordinators to do supervision at the health facilities.”* (District health manager, rural district)

Districts are required to submit quarterly reports to zonal NHIF officers on CHF membership and matching grant claims, which are then submitted to the national NHIF office. The NHIF has modified the requirements for matching fund claims, to reduce the risk of fraud (Table 3). Previously, there was no system to verify that the reported number of CHF members (and corresponding premium levels collected) was accurate, hence, districts could theoretically overestimate membership levels to obtain higher matching grants. Under the new system, district CHF coordinators must submit names of all CHF member household heads along with proof of revenue received. Many districts have never claimed for the matching grant and are now being encouraged to do so and can claim for the period since the start of the CHF. A computerised system is also being prepared by the NHIF that will facilitate future data capture and generate a database of members.

**Table 3:** Overview of matching fund claiming procedures before and after the reform

	**Pre-reform**	**Post-reform**
Frequency	Claims can be submitted at any time before end of financial year. Payments are made quarterly.	Claims should be submitted quarterly.
Procedure	District must enter into contract with the MoHSW.	As before, plus a list of names of all household head CHF members and their dates of joining. District must enter into contract with the NHIF. Plus:
	Provide bank statement and reconciliation and summary of CHF revenue and expenditure.	A letter which has been signed by the District Executive Director (DED) /District Medical Officer (DMO).
		Matching grant request form.
		Copy of the cash books.
		A report of the CHF progress from the last fund request
		Government exchequer receipt.
Stakeholder involvement	District CHF coordinator prepares claim and submits to national CHF coordinator.	CHF coordinator prepares claim and submits to zonal NHIF manager.
Rules	Must have collected a minimum of USD 3,125 in order to submit a claim.	No minimum amount of funds are needed for those have already started receiving funds.
		Districts which are requesting matching fund for the first time have must have collected a minimum of USD 3,125.
Verification procedure	No formal verification procedure.	Some verification of claims.
Fund management	MoHSW releases funds to districts which are budgeted for annually.	MoHSW gives funds to NHIF annually, who transfer money directly to district CHF account, or facility accounts.
Speed of fund disbursement	Funds disbursed within a minimum of 2 months.	Funds should be disbursed within 30 days of receiving a claim.

Source: CHF coordinators and NHIF management.

### Communication and awareness of the reform

There was very little media coverage of the reform in the first year following the MOU. Shortly after signing the MOU, the NHIF action plan was presented to the health financing technical working group at the MoHSW, briefing national level stakeholders of the reform. Some of the development partners working to support the CHF, such as the Swiss Agency for Development and Cooperation (SDC), German Technical Cooperation (GIZ), and the German Development Bank (KFW) were also consulted.

In February 2010, the MoHSW sent out a letter informing the districts that they should claim the matching grant from the NHIF. A similar letter was sent by PMO-RALG to the District Executive Officer (the head of the District Council) soon after. The district managers informed the members of the Council Health Management Team (CHMT) about the reform during their routine meetings, along with the CHSB and district CHF and NHIF coordinators. However, there was no clear strategy or guidelines for communicating the reform at the district level and below.

At the time of the first two field trips (May and August 2011) there was very little awareness of the reform outside of the district management. Health workers and health facility governing committee members only knew of the reform in relation to the matching fund claiming procedures.

*“We were not involved in this discussion but we were only informed and when we went to claim the matching fund for the CHF we were told that we should claim it from the NHIF not from the Ministry of Health.”* (District manager, rural district)

Generally, all stakeholder groups felt there had been insufficient information about the origin of the reform as of August 2011.

*“I think if we want these changes to be known […] the best way is training, seminars and other things that governments can prepare to inform people about this striking change.”* (HFGC member, rural district)

*“Communication was not enough from the national or regional level to inform the district level about the reform.”* (District health manager, urban district)

When researchers returned to the field in February 2012, there was a greater awareness of the reform within the district, resulting from the national meeting of CHF coordinators at the end of 2011 described above. However, there was no knowledge of the reform among NHIF or CHF members. Although one NHIF member had heard about the public awareness campaign for the CHF, they did not realise this was being conducted by the NHIF.

### Acceptability of the reform at the district level

Most district level respondents were favourable to the reform which was seen to improve efficiency.

*“Both are insurance schemes, I think they can support each other, for instance supervision can be done using NHIF zonal offices in one time and save money and time.”* (District manager, urban district)

*“When you look at the NHIF and CHF they are almost the same though their approach is different. Perhaps the Ministry of Health felt that it is better these two schemes work together so as to improve the services of the two schemes.”* (District manager, rural district)

However, one respondent reported that the new NHIF system for claiming matching funds has increased their workload and limited their capacity to claim.

“*The NHIF requires that when you are applying for matching fund you should attach receipts, a list of CHF members, bank statement, bank reconciliation, etc. This makes work very difficult and many people have failed to apply for a matching fund.*” (District manager, rural district).

At the facility level, committee members had high expectations that CHF members would get access to a wider range of services in line with NHIF members.

*“NHIF members, if there are no drugs at the facility, they go to town and get drugs from drug shops, also they get treated at all facilities, whereas CHF members here get treatment only at this dispensary. If that is the reform then we will also get access to other services like the NHIF people”* (HFGC, rural district)

*“If they will be administered by a single organ it will be good because […] they will try their best to improve health insurance [benefits], and not create differences [between schemes] of what we can get when we fall sick.”* (HFGC, urban district)

NHIF members also supported the single management of the two schemes. However, they felt that the revenue from each scheme should be kept separate.

*“[…] the need of having one management is good for these two schemes [which] have similar intentions, but financially they should operate independently.”* (NHIF member)

They did not support the use of NHIF funds to cross-subsidise benefit payment or the costs of administering the CHF, due to concerns about the financial sustainability of the CHF in light of unreliable member income, and a greater disease burden among poorer groups targeted by the CHF. They were reluctant for NHIF funds to be used to support better off CHF members, and thought the poor should be supported by the government.

*“There is a possibility that only salaries of the civil servants will be used to finance the services [of CHF members].”* (NHIF member)

*“[…] the government is increasing the burden of the NHIF to escape some of its responsibility.”* (NHIF member)

Some members felt concerned about management weaknesses in relation to meeting NHIF member needs, and thought the reform could overburden the NHIF. Most respondents emphasized the need first and foremost to prioritise NHIF members accessing quality services.

*“NHIF has a lot of problems, thus to add more responsibility is to increase more failure.”* (NHIF member)

Other community members interviewed had difficulty voicing opinions about the reform due to limited knowledge of the reform and the NHIF.

### Financial flows

**Figure 3:**
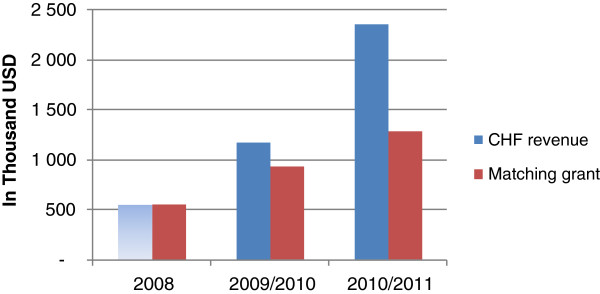
Overview of CHF revenue and matching grant payments between 2008–2011 in USD. SOURCE: [31]; [32]; [33]. Note that data were not available on CHF membership in 2008, hence it was not possible to estimate CHF revenue, only matching grant disbursements. 1. Ministry of Health and Social Welfare, Health Sector Public Expenditure Review, 2009/10. 2011: Dar es Salaam, Tanzania. 2. Ministry of Health and Social Welfare, Health Sector Public Expenditure Review, 2010/11 Draft,. 2012: Dar es Salaam, Tanzania. 3. MOHSW, Matching grand disbursement records. 2008.

National revenue from CHF member contributions has increased over time, with a steeper increase since the NHIF took over CHF than in the year preceding the takeover (Figure 3). However, total matching grant disbursements in relation to CHF revenue have reduced from 79% of revenue in 2009/2010 to 54% in 2010/2011, a likely result of the new claiming system introduced by the NHIF.

**Figure 4:**
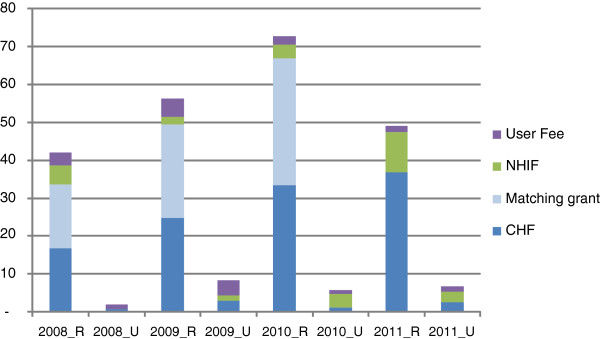
Overview of total district funds from NHIF reimbursements, CHF premiums and user fees in the two case study districts between 2008–2011 in thousand USD. NOTE TO TABLE: _R: RURAL; _U: URBAN Data Sources: Annual reports of revenue from the CHF coordinators in the respective districts. In the urban district, the CHF coordinator did not report on NHIF revenue. These data were obtained from the district NHIF coordinator.

Total funds from CHF, NHIF reimbursements and user fee revenue (hereafter referred to as cost sharing funds) remained a relatively constant and minimal share of the total resource envelope available to local government authorities accounting for around 4% of total expenditure between 2007/2008 and 2010/2011 [32,33]. However, in the two case study districts, total cost sharing funds had increased in absolute terms, as a result of the increase in CHF revenue since the reform (Figure 4). CHF revenue (inclusive of matching grant funds) increased from 74% of cost sharing revenue in 2008 to 93% in 2010 in the rural district (Figure 4). In 2011, although CHF revenue increased relative to previous years, the matching fund claim had not yet been submitted to the NHIF, reducing overall revenue. The increase in NHIF reimbursement funds in the rural district in 2011 is likely due to increased claims in that year, although it is also possible that increased visits to the districts resulting from the reform may also have had an effect. While CHF funds increased between 2008 and 2009 in the urban district, they remained very low. In parallel, reported user fee revenue decreased in the rural district from 5,000 USD in 2009 to 2,000 USD in 2010 and 2011, reflective of a reduction in the number of uninsured patients attending facilities. There was no evidence of sustained user fee reduction in the urban district.

**Figure 5:**
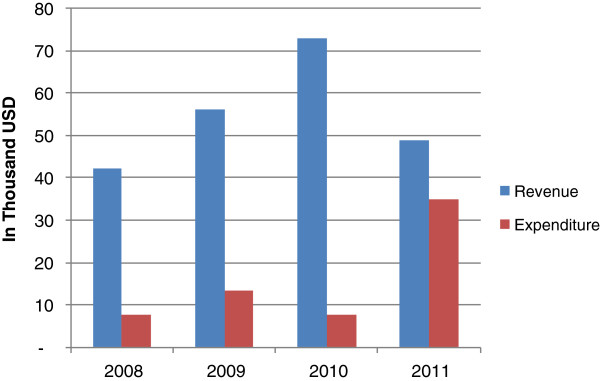
**Comparison of cost sharing revenue**^**b **^**and expenditure for 2008–2011 in the rural district in thousand USD.** SOURCE: [34], [35], [36].

The reform has had little effect on district expenditure of cost sharing funds. Expenditure of cost sharing revenue increased from 18% of revenue in 2008 to 24% in 2009, but fell again in 2010 to 11% in the rural district (Figure 5). However, in 2011 expenditure increased dramatically to 71% of revenue, a result of district efforts to sensitise facility in-charges on the importance of submitting claims to spend their revenue, not a result of the reform. In the urban district, expenditure data were not available.

The combined costs of treating CHF patients and administering the CHF at the facility level, exceeded revenue by an average of 538 and 567 USD per year in the rural and urban dispensaries, respectively; and by 2,556 USD and 3,322 USD per year in the rural and urban health centres, respectively, during the period 2008 to 2011 when using SHIELD data on utilisation to estimate treatment costs. However, the revenue outweighed the cost in the rural health centre when using facility registers to estimate treatment costs. The ratio of revenue to cost remained broadly constant over the period 2008 to 2011, suggesting that there were no gains in efficiency since the reform.

### Impacts of the reform on pooling and purchasing

Since the reform the number of districts that have introduced the CHF has increased from 92 to 111 between 2009 and 2011 [37]. At the end of 2009 about 43% of councils that had established CHF were not active (had no members) [38], this had been reduced by half by 2011 [37].

**Figure 6:**
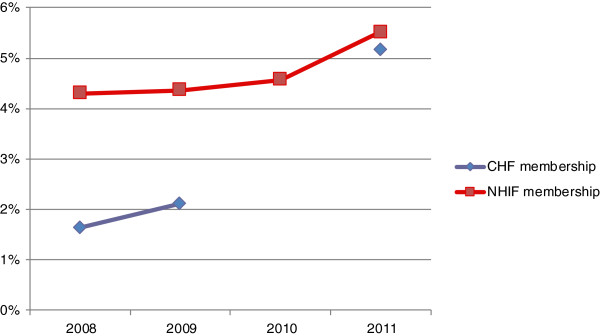
**Trends in health insurance coverage for the NHIF and CHFbetween 2008 and 2011.** SOURCE: NHIF data [20]; CHF data (MoHSW, 2008, NHIF 2009–2011), using projection-based population.

National CHF coverage increased from less than 2% to over 5% between 2008 and 2011 (Figure 6). In June 2011, the NHIF released funds to pay for CHF cards for the poor in a number of districts across the country, including the rural district. Although the effects of this will not yet be picked up in national figures, it is expected this will further enhance coverage over the coming years.

In many districts, CHF funds are still pooled at district level, allowing for cross subsidisation across health facilities. However, there has been no move to pool CHF funds at a higher level. Further, as more districts begin to open facility bank accounts, this could reduce the size of the risk pool, and limit cross-subsidisation, unless district accounts are maintained to allow for cross-subsidisation.

During the national meeting with CHF coordinators in late 2011, the importance of districts contracting with referral services to extend benefits available to CHF members was highlighted by NHIF managers, however, we cannot yet say whether this has resulted in extended benefits to members and in how many districts.

## Discussion

As a result of the reform to date, the CHF has been effectively embedded within the NHIF organisational structure, bringing more intensive and qualified supervision closer to the district. Reporting systems have been improved, generating routine data on coverage and matching grant claims and disbursements for NHIF and MoHSW use. The revisions to the matching grant claiming process should ultimately facilitate the district reporting process; however, at present they have increased the workload for district managers, and led to a reduction in the number of claims made and amount of revenue received by districts. Ensuring that districts are able to claim and receive matching funds in a timely manner is critical to the longer term sustainability of CHF, and it will be important to monitor this process over time.

Since the takeover of the CHF, national membership has more than doubled. It is likely that the improved supervision and reporting systems along with the national CHF information campaign played a critical part in the increase. District managers are likely to have felt more motivated to increase coverage as a result of closer monitoring. NHIF now offer some degree of quality assurance to CHF members, and by supporting districts to contract with referral facilities promise to offer a wider range of services, also making the scheme more attractive to prospective members.

There are clearly a range of other factors accounting for increased CHF coverage. The difference in CHF coverage and revenue trends between the two case study districts illustrates the important role of district management, with the rural district taking the initiative to introduce a range of innovations to increase coverage independently of the NHIF. In this respect, the NHIF could play an important coordination role by sharing information on best practices across districts to enhance performance among struggling districts.

Although the reform process is clearly ongoing and it is still at an early stage, initial levels of awareness were low due to the reform’s conceptualisation at the central level and top down implementation, and limited initial diffusion below district level. Awareness is, however, on the increase as a result of the recent national meeting with CHF coordinators and an intensified national information and communication campaign. Implementation was also delayed and the action plan revised based on fund availability.

Although it is expected that CHF legislation will be modified in subsequent phases of the reform, no attempt was made to do so within this first phase. As a result, no changes to the CHF design or district management structures have so far been possible. Further, the NHIF are not yet embedded within district management structures, as the CHSB does not include NHIF staff.

Key aspects of scheme management to date remain in the hands of district officials who often have other full time activities (a problem noted elsewhere [39]), and the burden of enrolling and sensitising members remains entrusted to facility managers and village leaders. In districts without facility bank accounts this limits facility capacity to spend the money resulting in many facilities consuming only a fraction of the resources they generate, which may demotivate staff from increasing enrolment rates, and increase community drop out, as quality of care does not improve. While the introduction of facility bank accounts could go some way towards addressing this problem (by making funds directly available to facilities), it can also result in a fragmented risk pool, unless a portion of funds are retained at district level for cross-subsidisation. Furthermore, operating bank accounts places additional responsibilities on health workers who typically do not have accounting skills. We were unfortunately unable to assess levels of expenditure by facilities with bank accounts. However, even if districts were able to fully utilise the CHF funds generated, if premiums remain at current levels, the revenue generated cannot offset the treatment and administrative costs of the CHF, meaning that the scheme will run at a net loss. The reform has not, to date, affected premium levels which also remain under the control of the district council.

One of the reported objectives of merging insurance schemes is to reduce administrative costs. However, due to the limited national level management systems prior to the reform, there is no evidence that the reform has in fact so far reduced administrative costs. Rather, the reform has, for the time being, resulted in the introduction of another level of administration (regional/zonal and national level), a larger number of national level management staff, and more intensive reporting requirements at district level. NHIF are also encouraging districts to employ full time CHF coordinators. Any rationalisation of administrative tasks at district level and below is impeded by the governing legislation underpinning the CHF. However, as there are no NHIF management structures below the regional level, ultimately district level management structures would be required which would impose additional costs.

A further challenge facing the reform is the unequal benefit package for members of the respective schemes and the lack of alignment between provider payment mechanisms for referral care. The failure to assure such alignment could lead to providers either refusing to enter into a contract with the CHF, or favouring NHIF over CHF members for the same services [40]. A critical issue regarding the future acceptability of the reform will be the handling of expectations on benefit harmonisation. It will therefore be important to monitor acceptability to key groups as the reform evolves.

The study suffered from a number of limitations. The field work took place two years after the reform, and little change had been observed at district level at the time of the study; therefore, some respondents found it difficult to form views on the reform and its acceptability. Reforms of this kind often take time to lead to concrete changes on the ground and the current assessment serves as an initial indication of progress and the initial implementation process rather than a definitive evaluation of impact. Clearly ongoing assessment of the reform will be important to ascertain longer term effects as the reform tackles management structures at district level and ultimately the design of CHF. Furthermore, monitoring financial flows at district level proved challenging as such data were often missing, incomplete, or erroneous. The problem was especially acute in the urban district where facilities have their own bank accounts, and the CHF coordinator lacked regular reports on revenue and had no information on expenditure.

However, a number of lessons can be drawn from this study for other countries planning to link informal and formal sector insurance schemes. Informal schemes can clearly benefit immensely from the experience of formal sector schemes in terms of improved data systems, fraud prevention, and supervision. There is limited international evidence of the use of the administrative and management functions of a formal sector insurance scheme to support an informal sector scheme. Such functions are typically outsourced to professional organisations [40]. However, the Tanzanian example demonstrates that informal sector schemes can benefit from the support of management and reporting systems from formal sector insurance schemes, especially in cases where informal sector schemes are run by Ministries of Health or small non-governmental organisations that may lack health insurance expertise.

However, effects will be maximised if legal frameworks underpinning schemes can be harmonised early on. Further, it is questionable whether such mergers will reduce administrative costs when they result in the creation of new layers of management/administration and when formal sector schemes lack management systems at the lower levels of the health system which are critical to administering community insurance schemes. Further research quantifying the impact of such reforms on administrative costs should be encouraged.

## Conclusions

The Tanzanian experience indicates that informal sector schemes, such as the CHF, can benefit from merger with formal sector schemes through improved data systems, supervision, and management support. Health insurance coverage has increased since the reform, especially among the informal sector. Although the evidence is limited, it is also possible that user fees are reducing as a result of insurance expansion in districts where CHF coverage is reasonably high. However, risk pools remain highly fragmented, and the opening of bank accounts at facility level in some districts risks further fragmentation. It will therefore be important to pay careful attention to equity. A reduction in administrative costs is not guaranteed. Overall the reform effects will be maximised if legal frameworks can be harmonised early on.

## End notes

^a^The aim of the SHIELD research project was to critically identify and evaluate existing inequities in health care in Ghana, South Africa, and Tanzania, and the extent to which health insurance mechanisms could address equity challenges.

^b^Cost sharing revenue includes: user fee revenue, community health fund contributions, and National Health Insurance Fund reimbursements.

## Abbreviations

CHF: Community health fund; CHSB: Council health service board; FGD: Focus group discussion; GIZ: German technical cooperation; HFGC: Health facility governing committee; KFW: German development bank; MOHSW: Ministry of Health and Social Welfare; MOU: Memorandum of understanding; NHIF: National health insurance fund; PMO-RALG: Prime Minister’s office for regional administration and local government; SDC: Swiss development cooperation; SHIELD: Strategies for health insurance and equity in less developed countries; USD: United States dollar.

## Competing interests

The authors declare that they have no competing interests.

## Authors’ contributions

JB conceptualized and designed the study, undertook data analysis and wrote the first full draft of the paper. StM undertook qualitative data collection and analysis, and helped to draft the manuscript. AK collected and analysed quantitative data and critically revised the paper. SuM collected quantitative data and critically revised the paper. JT collected and analysed quantitative data and critically revised the paper. GM contributed to the study design and critically revised the paper. MA collected qualitative and quantitative data and critically revised the paper. JM conceptualized and designed the study, undertook data analysis, helped draft the manuscript and critically revised the paper. All of the authors read and approved the manuscript.

## Authors’ information

JB is a Senior Lecturer at the London School of Hygiene & Tropical Medicine, and worked at the Ifakara Health Institute from 2007–2012. JB has a BA, an MSc in Health Economics and a PhD in Health Economics.

StM is a Lecturer at the University of Dar es Salaam. SM has a BA and an MA in Development Studies. He has a PhD in Public Health.

AK is a Research Scientist doing health financing research at the Ifakara Health Institute. AK has a BA and an MA in Economics.

SuM is a Research Officer doing health financing research at the Ifakara Health Institute. SuM has a BSc in Economics.

JT is a CHF coordinator and Health Secretary in the rural district. He has a Diploma in Health Administration.

GM is a Research Scientist doing health financing research at the Ifakara Health Institute. GM has a BA and an MA in Economics and a PhD in Health Economics.

MA is health financing coordinator at the Ministry of Health and Social Welfare. MA has a BA, an MBA and an MSc in Health Economics.

JM is a Research Scientist doing health financing research at the Ifakara Health Institute. JM has a BA and an MA in Sociology and a Post-graduate Diploma in Public Health.
